# Supplementary material for: Downregulation of LAMB3 Altered the Carcinogenic Properties of Human Papillomavirus 16-Positive Cervical Cancer Cells
Source: Int J Mol Sci. 2024 Feb 22;25(5):2535. doi: 10.3390/ijms25052535 (PMC10931834; doi:10.3390/ijms25052535)
Supplement: Supplementary file 1 [file ijms-25-02535-s001.zip › supplementary Figure 1.pdf]

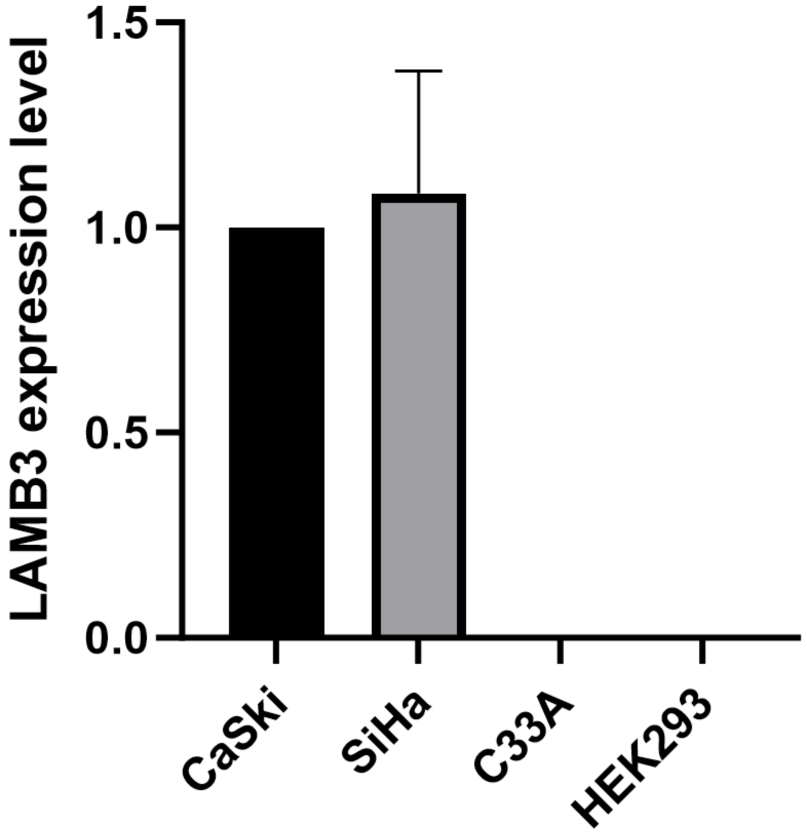

Figure S1. Relative LAMB3 mRNA expression levels in three cervical cancer cell lines including HPV16 positive cell lines (CaSki and SiHa) and HPV negative cell line (C33A) and Human Embryonic Kidney (HEK293), GAPDH was used as a housekeeping gene. The data was from three independent experiments
