# Supplementary material for: Downregulation of LAMB3 Altered the Carcinogenic Properties of Human Papillomavirus 16-Positive Cervical Cancer Cells
Source: Int J Mol Sci. 2024 Feb 22;25(5):2535. doi: 10.3390/ijms25052535 (PMC10931834; doi:10.3390/ijms25052535)
Supplement: Supplementary file 1 [file ijms-25-02535-s001.zip › Supplementary Table 4 and 5.pdf]

Supplementary Table 4. Percentage of cell cycle population in LAMB3 siRNA knockdown SiHa and CaSki cell lines.

% Gated	SiHa		CaSki	
	NTC	KDLAMB3	NTC	KDLAMB3
**G1 phase**	65.683 ± 3.650	57.1567 ± 0.871	54.207 ± 0.905	45.770 ± 0.440
**S phase**	7.317 ± 0.601	9.880 ± 0.498	10.740 ± 0.410	7.757 ± 0.556
**G2 and M phase**	11.823 ± 0.723	13.613 ± 0.750	14.373 ± 0.559	13.760 ± 0.616
**Sub G1 phase**	14.360 ± 1.951	18.420 ± 1.490	19.770 ± 1.785	31.250 ± 1.135

Supplementary Table 5. Percentage of apoptosis in LAMB3 siRNA knockdown SiHa and CaSki cell lines.

% Gated	SiHa		CaSki	
	NTC	KDLAMB3	NTC	KDLAMB3
**Live cells**	93.233 ± 0.447	90.250 ± 1.035	94.753 ± 1.019	94.053 ± 0.804
**Apoptotic cells**	6.767 ± 0.447	9.750 ± 1.035	5.247 ± 1.028	5.947 ± 0.804
